# CKM and TERT dual promoters drive CRISPR–dCas9 to specifically inhibit the malignant behavior of osteosarcoma cells

**DOI:** 10.1186/s11658-023-00464-7

**Published:** 2023-07-06

**Authors:** Yawei Hu, Hao Zhang, Zengfeng Guo, Jianhua Zhou, Wang Zhang, Ming Gong, Jiawen Wu

**Affiliations:** Department of Spine Surgery, People’s Hospital of Longhua, Affiliated Hospital of Southern Medical University, Shenzhen, China

**Keywords:** Osteosarcoma, CKM promoter, TERT promoter, CRISPR–dCas9 system, Gene therapy

## Abstract

Improvements in treatment and chemotherapy have increased the survival rate of osteosarcoma, but overall efficacy remains low, highlighting the need for new gene therapy methods. Clustered regularly interspaced short palindromic repeats–deactivated Cas9 (CRISPR–dCas9) technology offers a promising strategy, but targeting osteosarcoma cells precisely is a challenge. We designed a system to achieve specific expression of CRISPR–dCas9–KRAB in osteosarcoma cells by using the creatine kinase muscle (CKM) promoter to drive dCas9–KRAB and the telomerase reverse transcriptase (TERT) promoter to drive single guide (sg)RNA expression. We inhibited the *MDM2* proto-oncogene using this system in vitro, which efficiently inhibited the malignant behavior of osteosarcoma cells and induced apoptosis without affecting normal cells. In vivo experiments demonstrated that this system effectively inhibited the growth of subcutaneously transplanted tumors in nude mice. These findings provide a new method for precise identification and intervention of osteosarcoma with significant implications for the development of gene therapy methods for other cancers. Future research should focus on optimizing this system for clinical translation.

## Introduction

Osteosarcoma originates from mesenchymal tissue and mostly occurs in bone tissue [1]. It is a malignant bone tumor commonly found in adolescents or children [2]. Males are significantly more affected than females. It has rapid progression, early lung metastasis, poor prognosis, easy recurrence, and even death. The current treatment of osteosarcoma includes complete resection of the lesion by surgical means and comprehensive adjuvant antitumor therapy in the perioperative period [3]. Neoadjuvant chemotherapy, as the first choice for chemotherapy, can not only increase the safety of surgery, but also prepare suitable prostheses for patients. However, the current chemotherapy drugs are limited, often accompanied by obvious systemic side effects, which bring pains to patients [4]. There are still many difficulties and doubts in the treatment of osteosarcoma. Therefore, it is imperative to find more effective and less toxic treatment methods and to clarify their mechanism of action.

In the past 10 years, gene therapy, immunotherapy, and molecular targeted therapy of tumors have become the focus of current clinical osteosarcoma research, and exploring new and more effective treatment methods has become an important direction of osteosarcoma research [5]. The tumor suppressor genes in normal cells are easy to transform into tumor cells to replace defective genes, thereby inhibiting tumor growth or reversing its phenotype. This replacement therapy has positive effects on tumor treatment [6]. The tumor suppressor genes currently tested for gene therapy include *p53*, *p16*, *Rb*, *p21*, and so on [7]. Another approach is to block the expression of tumor-associated proto-oncogenes, thereby inhibiting tumor growth. The abnormally activated proto-oncogenes in osteosarcoma include *MDM2*, *SAS*, *c-myc*, and so on [8]. The *MDM*2 oncogene was successfully cloned in 1992, and its mutation and amplification have been identified in a variety of tumors [9]. Changes in the *MDM2* oncogene are of great significance for elucidating the mechanism of tumor pathogenesis and metastasis, as well as having clinical significance.

The clustered regularly interspaced short palindromic repeats–Cas9 (CRISPR–Cas9) system provides a powerful technical means for targeted gene editing [10]. Guided by sequence-specific single guide (sg)RNAs, the CRISPR–Cas9 system can precisely introduce double-stranded nicks at the exact location of the target DNA. Compared with existing gene editing methods, this system has superior simplicity, specificity, and effectiveness [11]. At present, a large number of CRISPR–Cas9 gene editing studies involving multiple species have fully demonstrated the great potential of this technology, which is a promising tool for disease treatment research and the clinical applications of this technology are very promising [12]. The CRISPR–Cas9 gene editing technology has great potential in tumor gene therapy. At present, animal and clinical studies on this technology mainly include two methods: directly attacking key genes of cancer cells and editing immune cells [13]. Some technologies have entered the stage of clinical trials, but this technology also has problems such as off-target phenomenon and major ethical dilemmas.The long-term consequences of genetic alterations made through this technology are still largely unknown, and the ethical implications of manipulating the human genome are complex and far-reaching.

The nuclease cleavage activity of Cas9 depends on two domains: RuvC and HNH, which are responsible for cleaving both strands of the DNA strand [13]. These two conserved endonuclease domains could be mutated: aspartic at position 10 of the RuvC catalytic domain was mutated to alanine (D10A) and histidine at position 840 of the HNH catalytic domain was mutated to alanine (H840A). Cas9 protein loses endonuclease activity, and the inactive Cas9 is called dead Cas9 (dCas9) [14]. Although dCas9 has lost the ability to cut DNA, it can still bind to specific DNA sequences under the guidance of gRNA. The dCas9–sgRNA complex acts as a scaffold to recruit a series of effector molecules to specific locations to activate gene transcription or prevent RNA polymerase from binding to the promoter and inhibit transcription elongation due to steric hindrance, thereby having a regulatory function [15]. At present, dCas9-based CRISPR interference (CRISPRi) systems have been validated in a variety of model organisms, but their ability to repress gene transcription is low in eukaryotes. To improve the strength of transcriptional repression, dCas9 was fused and expressed with different transcriptional repressors and verified [16]. Finally, it was found that the dCas9–Krüppel-associated box (KRAB) fusion protein has a high repression efficiency as KRAB is a very strong transcriptional repressor domain. When fused with the dCas9 protein, the resulting dCas9–KRAB fusion protein can effectively silence gene expression in eukaryotes by recruiting histone deacetylases and other chromatin-remodeling factors to the target gene promoter. The development of dCas9–KRAB-based CRISPRi systems with high transcriptional repression efficiencies has opened up new avenues for genetic engineering and gene regulation in eukaryotes.

In recent years, a lot of experimental research in tumor gene therapy has studied the CRISPR–dCas9 system, but there are still some problems in the targeted expression of this system in tumor cells [17]. Successful gene therapy in vivo requires that the therapeutic gene can be specifically delivered to target cells, and the expression of the therapeutic gene needs to be restricted to malignant cells to avoid nonspecific cytotoxicity [18]. At present, there are several methods to specifically express therapeutic genes in tumor cells, including the use of tissue or tumor cell-specific promoters to regulate the targeted expression of therapeutic genes in malignant cells without affecting surrounding normal cells [19]. Creatine kinase muscle (CKM) encodes a muscle-specific isozyme, which is one of the muscle-specific genes activated in the terminal differentiation of skeletal muscle, and is a marker of muscle development and differentiation [20]. CKM can combine with the M-band of myofibrils and is a key enzyme in energy metabolism of skeletal muscle. In addition, CKM is also an effective substrate of protein kinase c, which is an important component in the signaling pathway of cell growth. Human and mouse studies have shown that the CKM promoter is specifically expressed in muscle tissue, and it has been widely used in transgenic and gene therapy. This promoter only has activation properties in skeletal muscle, but has no transcriptional activity in various cells except skeletal muscle. Since the gene promoter only specifically initiates the expression of downstream genes in skeletal muscle, it can meet the requirement of specific expression of target gene in skeletal muscle. Telomeres are nuclear protein structures at the ends of chromosomes that maintain genome stability and prevent cancer. While cancer cells can replicate tumor cells by activating the telomere maintenance mechanism, activation of telomerase through abnormal expression of telomerase reverse transcriptase (TERT) is the most prevalent telomere maintenance mechanism in cancer [21]. The TERT promoter has no obvious transcriptional activity in normal cells, but has a high transcriptional activity in cancer cells, indicating that the TERT promoter can specifically regulate the expression of target genes only in cancer cells [22].

Because the CRISPR–dCas9–KRAB expression system includes two components, the CRISPR–dCas9–KRAB protein and sgRNA, we hypothesized that the specific expression of CRISPR–dCas9–KRAB in osteosarcoma cells could be achieved by using the CKM promoter to drive dCas9–KRAB and the TERT promoter to drive the expression of sgRNA. In this study, we designed sgRNA targeting the *MDM2* proto-oncogene and specifically inhibited the expression of *MDM2* gene in osteosarcoma cells using the CKM and TERT dual promoter-driven CRISPR–dCas9–KRAB system. In vitro and in vivo experiments show that this system can effectively inhibit the malignant biological behavior of tumors, thus providing a potentially effective new tool for gene therapy of osteosarcoma.

## Materials and methods

### Cell culture

The human osteosarcoma cell lines MG-63 (cat. no.TCHu124), Saos-2 (cat. no.TCHu114), and U-2 OS (cat. no. SCSP-5030), and the normal human osteoblast cell line hFOB 1.19 (cat. no. GNHu14) were purchased from the Cell Bank of the Chinese Academy of Sciences (Shanghai, China). Fetal bovine serum (FBS) was purchased from HyClone (Utah, USA) and RPMI-1640 medium was purchased from GE Healthcare Life Sciences (Pennsylvania, USA). Cells were cultured in RPMI-1640 containing 1% penicillin–streptomycin and 10% FBS (complete medium), respectively. Complete medium was used and changed every 2 days until the number of attached cells reaches 75–90% in the dish in an incubator at 37 °C and 5% CO_2_. The desired number of cells was used for other experimental studies.

### Construction of plasmids expressing CRISPR–dCas9 driven by dual promoters

The promoter region of the *CKM*/*TERT* gene was amplified from the genome of HEK-293 cells by PCR and inserted into the dCas9–KRAB plasmid, which was a gift from Dr. George Church’s lab (Addgene plasmid #110,820; http://n2t.net/addgene:110820; RRID:Addgene_110820). The CKM promoter was used to drive the expression of the dCas9–KRAB fusion gene, while the TERT promoter was used to express the sgRNA. The sgRNA targeting and inhibiting the promoter region of the human *MDM2* gene was designed by CRISPR-ERA online software, and its sequence is: 5′-GCGATTGGAGGGTAGACCTG-3′.

### Real-time qRT–PCR

Total RNA was isolated from cells using Trizol reagent (Invitrogen), followed by reverse transcription to synthesize cDNA template. Next, qRT–PCR was performed using the cDNA template and specific PCR primers. The change in the amount of the amplification product in each PCR amplification reaction cycle was detected in real time by the change of the fluorescent signal, and finally the starting template was accurately and quantitatively analyzed. The GAPDH gene was selected as the internal reference, and the relative expression of the gene was calculated by the 2^−ΔΔCT^ method.

### CCK-8 assay for assessing cell proliferation

Cell Counting Kit-8 (CCK-8) reagents were purchased from Dojindo Molecular Technologies (Tokyo, Japan). Osteosarcoma cells and normal cells in logarithmic growth phase were selected under a microscope. The original medium was discarded and washed with phosphate-buffered saline (PBS). After trypsinization, the cell pellet was resuspended in complete medium. Then, 1 × 10^4^ cells at 100 μL per well were counted and transferred to a 96-well plate. After culturing in an incubator (37 °C, 5% CO_2_) for 24 h, the cells were attached to the bottom of the 96-well plate, and the experiments were carried out according to the reagent instructions. The experiment was repeated at least three times, and each group was set with three experimental wells. Optical density (OD) was measured at 450 nm wavelength.

### Wound-healing assay

A wound-healing assay was used to determine the effect of CRISPR–dCas9–KRAB on migration of several osteosarcoma and normal cell lines. Cells were seeded in six-well culture plates (5 × 10^5^ cells per well) until cell confluency reached 80%. Then, the medium was removed, and a “wound” is created. After two washes with PBS, wound images were captured at the time of injury (T0). After 24 h, pictures were taken of the same area and the size of the wound was measured, which was used to calculate the cell migration rate to estimate the migratory capacity of the cells.

### Apoptosis detection using Annexin V-FITC/PI staining

The adherent cells at the bottom were made into a cell suspension in a trypsinization vessel. After the suspension was centrifuged at 1.0 × 10^3^r/min for 7 min, the cells were washed three times with PBS. The next steps were performed according to the instructions of the Annexin V-FITC Apoptosis Kit. Cells were incubated in the dark for 15 min at room temperature and analyzed by flow cytometry.

### Transwell detection of cell invasion ability

After starvation of cells, appropriate dilutions of Matrigel were applied to the upper chamber and allowed to polymerize overnight. The chambers were plated with medium containing 20% FBS. Cells were trypsinized to prepare cell suspensions. The upper chamber was placed in the well plate, and cells were plated in the upper chamber after cell counting. Cells were incubated for 48 h, and then Matrigel in the upper chamber was wiped off with a cotton swab. Cells were fixed with 4% paraformaldehyde for 20 min. Finally, pictures were captured after staining with crystal violet or Giemsa.

### Nude mouse tumorigenesis experiment

In vivo experiments were performed in accordance with standard experimental guidelines and were accredited by the laboratory animal department and approved by the hospital ethics committee. Randomly selected male BALB/c nude mice (*n* = 6) were included in each experimental group. Cells were diluted to 2 × 10^7^ cells/ml using serum-free medium. Then, 200 µL of the cell suspension was injected subcutaneously into the back of nude mice to generate solid tumors. When the solid tumor grew to 200 mm^3^, the nude mice were randomly divided into the control group and the treatment group. Then, we measured the body weight and tumor size of nude mice every 3 days, and the tumor tissue was removed after 21 days for weighing and cryopreservation. Tumor size was calculated as follows: tumor size = (L × W^2^)/2 (where L is the length of the tumor tissue and W is the width).

### Statistical methods

SPSS 20.0 software was used to process the data. According to the experimental group, two groups of data were compared by a *t*-test, and multiple groups of data were compared by ANOVA. Values of *P* < 0.05 were considered statistically significant.

## Results

### Design and construction of CRISPR–dCas9 driven by dual promoters

**Fig. 1:**
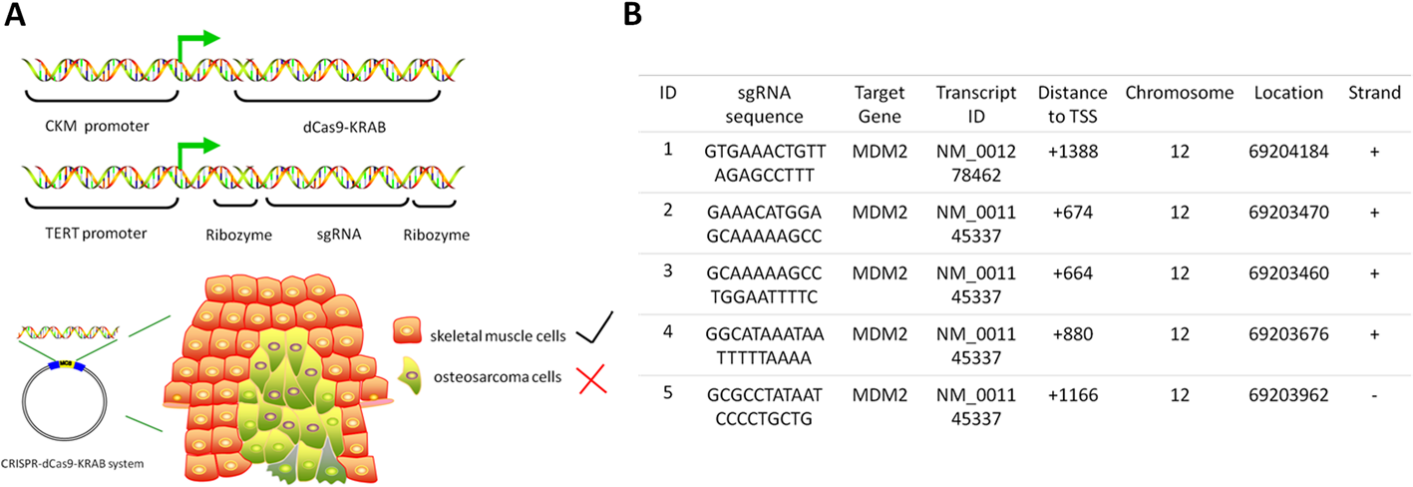
Design and construct of a CRISPR–dCas9–KRAB system driven by dual promoters. **A** Schematic diagram of the construction of CRISPR–dCas9–KRAB plasmids driven by CKM promoter and TERT promoter, respectively. This system exerts specific tumor-killing effects only in osteosarcoma cell lines. **B** Locations and sequences of five different sgRNAs targeting the repressed *MDM2* gene

Through genetic modification of traditional plasmids, we first tried to construct a CRISPR–dCas9–KRAB expression system co-regulated by dual promoters. We amplified the human CKM promoter and TERT promoter and placed them upstream of the dCas9–KRAB and sgRNA expression sequences, respectively, to replace the original persistent promoters including CMV and U6. To ensure that the sgRNA can stay in the nucleus and play its due targeting role, we inserted ribozyme sequences at both ends of the sgRNA (Fig. 1A). The sgRNA was designed to target the promoter region of the human *MDM2* gene (located downstream of the transcription start site) and to transcriptionally repress the expression of *MDM2* (Fig. 1B). We totally designed five different sgRNAs and selected the best performing sgRNAs for follow-up experiments. In our concept, the CKM promoter has a certain transcriptional activity and drives the transcription of dCas9–KRAB only in skeletal muscle cells. Similarly, only in tumor cells can the TERT promoter have relatively high transcriptional activity and drive sgRNA expression. Therefore, only in osteosarcoma cells can the two promoters have relatively high transcriptional activity at the same time, and then the sgRNA can cooperate with dCas9–KRAB to transcriptionally inhibit the expression of *MDM2*.

### CRISPR–dCas9 driven by dual promoters specifically inhibited *MDM2* expression in osteosarcoma cells

**Fig. 2:**
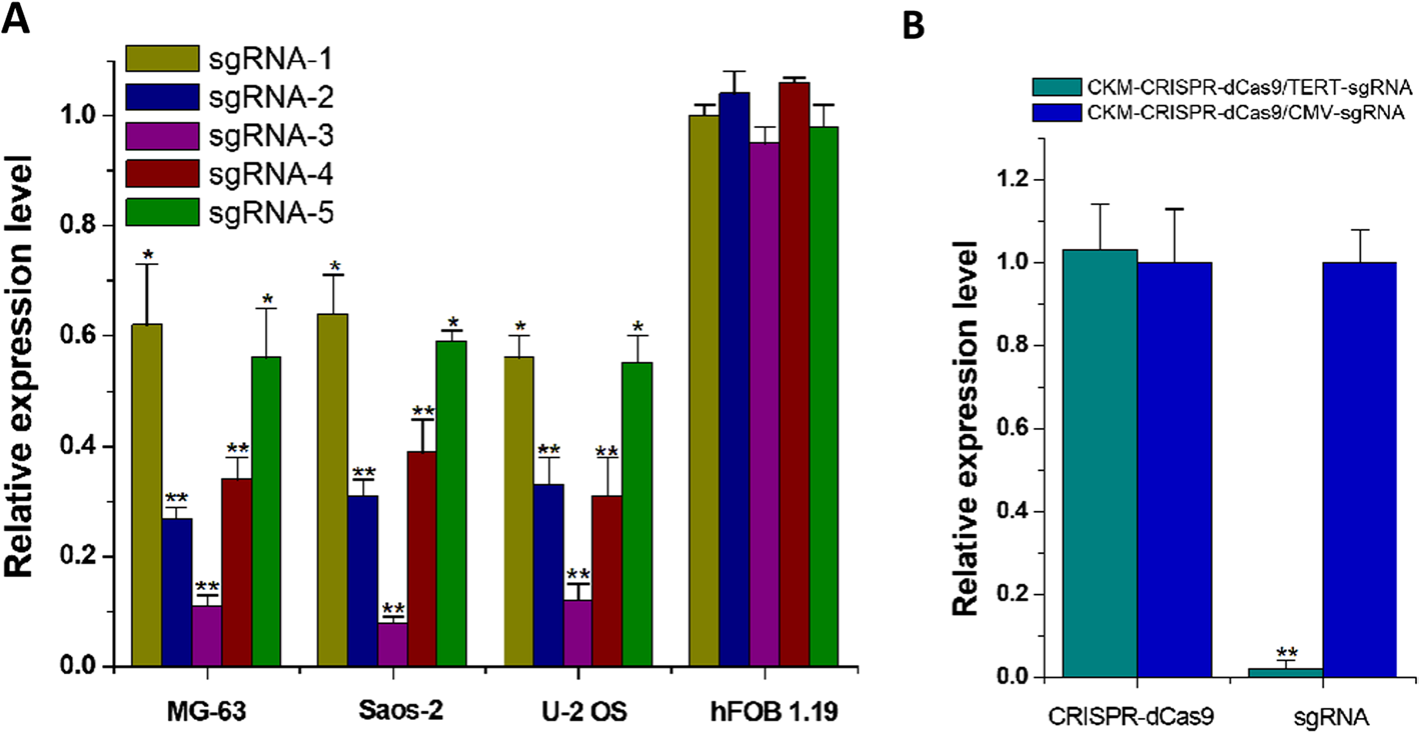
The CRISPR–dCas9–KRAB system under the control of bispecific promoters acts selectively in osteosarcoma cell lines. **A** The relative expression of *MDM2* gene in different cell lines transfected with CRISPR–dCas9–KRAB was detected by qRT–PCR. *P < 0.05, **P < 0.01. **B** The relative expression levels of CRISPR–dCas9 and sgRNA were detected by qRT–PCR in the normal cell line, respectively. **P < 0.01

To verify whether CRISPR–dCas9 driven by dual promoters has osteosarcoma targeting specificity, we transfected the constructed plasmids expressing the CRISPR–dCas9–KRAB system into osteosarcoma cells including MG-63, Saos-2, and U-2 OS, respectively, and normal osteoblasts hFOB 1.19. Forty-eight hours after transfection, qRT–PCR showed that the five sgRNAs inhibited the expression of *MDM2* to varying degrees in osteosarcoma cells. Among them, sgRNA-3 had the best effect, with an average silencing effect of 90%. In normal osteoblast hFOB 1.19, all sgRNAs could not significantly inhibit the expression of *MDM2* (Fig. 2A). Then, we detected the expression levels of CRISPR–dCas9 mRNA and sgRNA, and found that CRISPR–dCas9 could be expressed in normal cells, but the sgRNA expression level was almost undetectable (Fig. 2B). This should be due to the low activity of the TERT promoter in normal cells. The above results suggest that CRISPR–dCas9 driven by dual promoters has good targeting specificity for osteosarcoma.

### The inhibitory effects of CRISPR–dCas9 driven by dual promoters on osteosarcoma cell proliferation

**Fig. 3:**
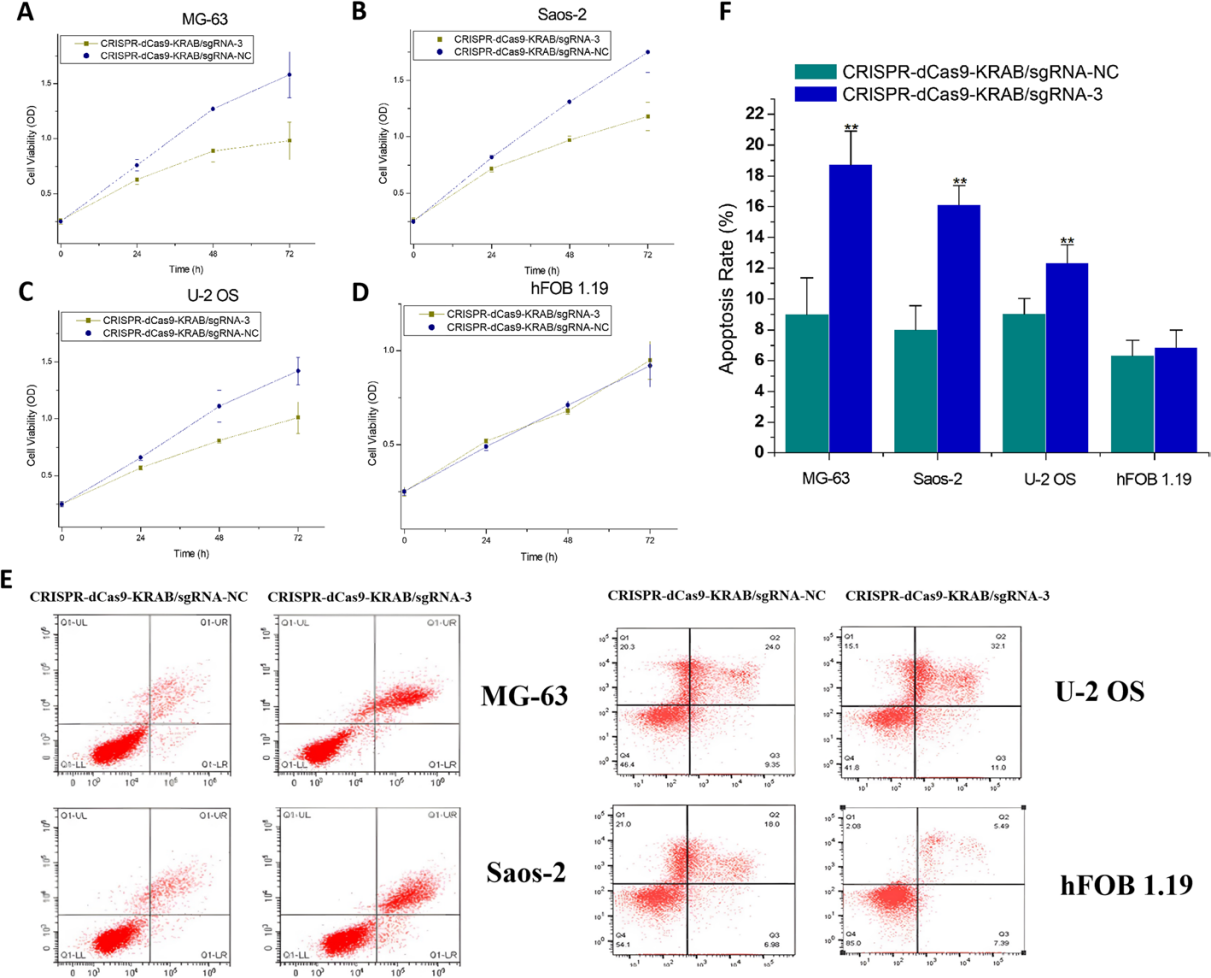
Effects of the CRISPR–dCas9–KRAB system on cell viability after 24, 48, and 72 h compared with the sgRNA control group. CCK-8 was used to detect cell proliferation viability. There were statistical differences between the two groups in **A**–**C**, and the *P*-value was less than 0.01. There was no statistical difference between the two groups in **D**. ANOVA was used for statistics. Flow cytometry was used to detect cell apoptosis rate (%). There were statistical differences between the two groups in MG-63, Saos-2, U-2 OS, and hFOB 1.19. The *P*-value was less than 0.01. ANOVA was used for statistics. **E** Representative pictures of apoptosis. **F** Statistical histograms

The impacts of CRISPR–dCas9 driven by dual promoters on the growth of osteosarcoma cells were observed through the detection of cell proliferation. As shown in Fig. 3A–D, after transfection of plasmids expressing the CRISPR–dCas9–KRAB/sgRNA-3 system for 24 h, the viability of osteosarcoma cells including MG-63, Saos-2, and U-2 OS were obviously inhibited compared with that in the negative control sgRNA group, and after 48 and 72 h, the viability of the cells was significantly inhibited. In contrast, the CRISPR–dCas9–KRAB/sgRNA-3 system had no significant effect on the proliferation behavior of normal cells hFOB 1.19, which was consistent with the results of qRT–PCR. The above results indicate that CRISPR–dCas9 driven by dual promoters can significantly inhibit the proliferation of osteosarcoma cells without affecting normal cells.

### The induction effects of CRISPR–dCas9 driven by dual promoters on osteosarcoma cell apoptosis

To further clarify whether the inhibitory effect of dual-promoter-driven CRISPR–dCas9 on cell proliferation is caused by apoptosis, we then used flow cytometry to analyze osteosarcoma cells transfected with the CRISPR–dCas9–KRAB/sgRNA-3 system overexpression plasmid, and the apoptosis level was detected. As shown in Fig. 3E and F, the percentage of apoptosis in osteosarcoma cells including MG-63, Saos-2, and U-2 OS was significantly higher than that in the negative control group at 48 h after plasmid transfection. In contrast, the CRISPR–dCas9–KRAB/sgRNA-3 system had no significant effect on the apoptosis level of hFOB 1.19 in normal cells, which was consistent with the results of qRT–PCR. The above results indicate that the dual-promoter-driven CRISPR–dCas9 can significantly promote the apoptosis of osteosarcoma cells without affecting normal cells.

### The inhibitory effects of CRISPR–dCas9 driven by dual promoters on osteosarcoma cell migration

**Fig. 4:**
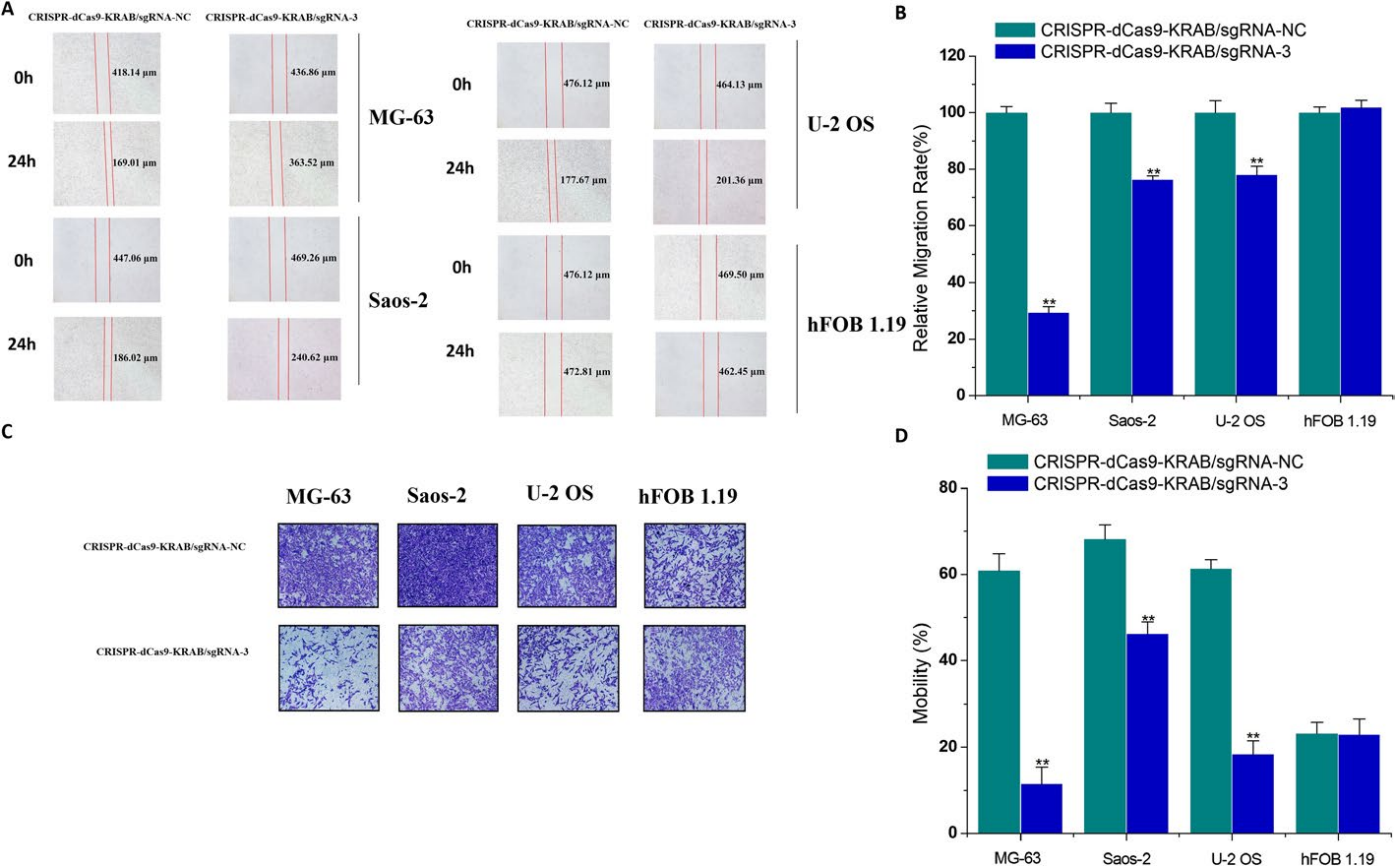
Effects of the CRISPR–dCas9–KRAB system on cell migration and invasion 24 h after transfection compared with the sgRNA control group. A wound healing assay was used to detect relative cell migration rate (%). There were statistical differences between the two groups in MG-63, Saos-2, U-2 OS, and hFOB 1.19. The *P*-value was less than 0.01. ANOVA was used for statistics. **A** Representative pictures of migration. **B** Statistical histograms. A transwell assay was used to detect relative cell mobility rate (%). There were statistical differences between the two groups in MG-63, Saos-2, U-2 OS, and hFOB 1.19. The *P*-value was less than 0.01. ANOVA was used for statistics. **C** Representative pictures of invasion. **D** Statistical histograms

We next tested whether specific expression inhibition of *MDM2* could suppress the motility of osteosarcoma cells. The effects of dual-promoter-driven CRISPR–dCas9 on the migration and invasion of osteosarcoma cells were observed by cell scratch and transwell experiments. As shown in Fig. 4A–D, 20 h after transfection of plasmids expressing the CRISPR–dCas9–KRAB/sgRNA-3 system, the migration and invasion behavior of osteosarcoma cells including MG-63, Saos-2, and U-2 OS was significantly inhibited compared with the negative control group. Similar to proliferation and apoptosis, the CRISPR–dCas9–KRAB/sgRNA-3 system had no significant effect on the migration and invasion behavior of normal hFOB 1.19 cells, which is consistent with the above results. These results demonstrate that dual-promoter-driven CRISPR–dCas9 can significantly inhibit the motility of osteosarcoma cells without affecting normal cells.

### The effects of CRISPR–dCas9 driven by dual promoters on the growth of tumor xenografts

**Fig. 5:**
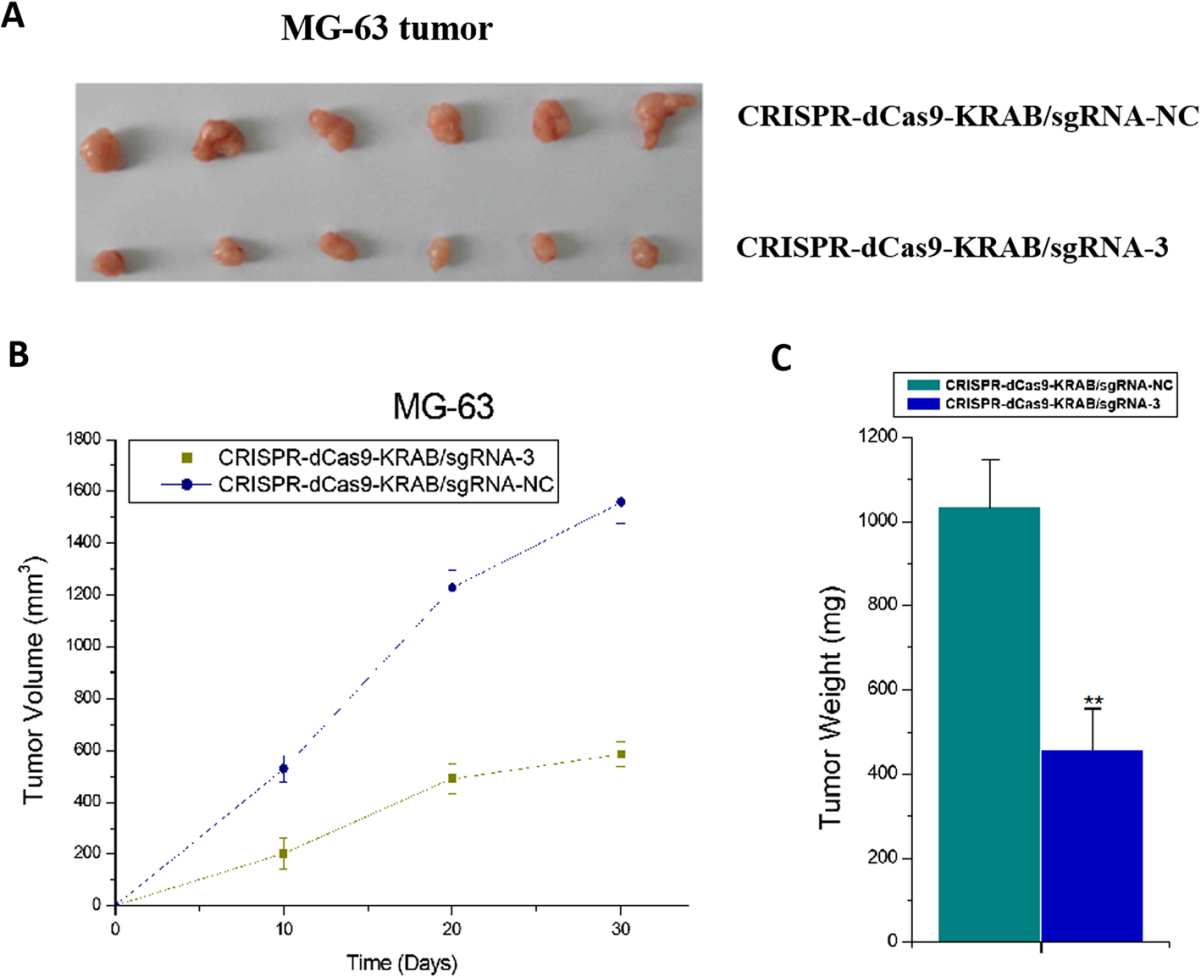
Effects of the CRISPR–dCas9–KRAB system on in vivo tumor cell growth compared with the sgRNA control group. A nude mouse tumorigenesis experiment was used to detect the relative cell growth. There were statistical differences between the two groups in MG-63. The *P*-value was less than 0.01. A Student’s *t*-test was used for statistics. **A** Representative pictures of tumors. **B** Statistical curves. **C** Statistical histograms

Finally, we tested whether the above in vitro results could be reproduced at the in vivo level. The in vivo inhibitory effect of dual-promoter-driven CRISPR–dCas9 on osteosarcoma was evaluated by constructing a subcutaneous transplantation model of nude mice inoculated with MG-63 tumor cells. Tumors in nude mice treated with lentiviruses overexpressing CRISPR–dCas9 were much smaller in size and weight compared with those in the sgRNA-negative control group (Fig. 5A–C). The above results indicated that dual-promoter-driven CRISPR–dCas9 could significantly inhibit the growth of osteosarcoma in vivo.

## Discussion

Cancer is a refractory disease with high mortality and worldwide concern. Malignant tumors cause one in six deaths worldwide, thereby threatening the lives of thousands of people [23]. Despite many exciting results in the field of cancer treatment, including surgery, radiotherapy, chemotherapy, targeted biological therapy, and novel combination therapies, high postoperative recurrence rates, radiotherapy/chemotherapy resistance, and harmful toxic and side effects of the disease are still obstacles to the survival time and quality of life of patients [24].

Research shows that cancer is a potentially fatal disease that accumulates altered expression of multiple genes and alters epigenetics across the genome [25]. Alterations in gene expression profiles in cancer often drive cancer progression and affect tumorigenesis. Over the past two decades, high-throughput transcriptome sequencing technologies have identified a large number of gene expression changes associated with cancer initiation and progression. Based on these advances, gene editing technology is expected to treat cancer by regulating gene expression and correcting gene mutations, which may lead to further breakthroughs in the field of precision medicine [26].

Various technologies, including zinc finger endonucleases (ZFNs), transcription activator-like effector nucleases (TALENs), and CRISPR–Cas systems, are used to achieve gene editing and transcriptional regulation [27]. The CRISPR–Cas system has the advantages of simple design, rapid implementation, low cost, and strong scalability. Researchers consider it to be a revolutionary gene editing toolbox that has been extended to almost all genomic targets. In particular, the system has been widely used in cancer research and has emerged as a potential method for cancer diagnosis and treatment.

In this work, we have presented a CRISPR–dCas-based transcriptional technology that shows promising potential for gene therapy in osteosarcoma. Our approach specifically targets the *MDM2* proto-oncogene using the CKM and TERT dual promoter-driven CRISPR–dCas9–KRAB system, resulting in a significant inhibition of malignant biological behaviors of osteosarcoma cells, without affecting normal cells.

Comparing our results to previous studies [28–30], we found that our approach provides a more targeted and specific gene therapy strategy for osteosarcoma. However, it is important to note that there are still limitations to our approach, including the potential for off-target effects, and further optimization is necessary to improve its efficacy and specificity.

Future research in this area should focus on addressing these limitations, including improving the delivery of the CRISPR–dCas9–KRAB system to osteosarcoma cells, optimizing the specificity of the system, and exploring potential toxic side effects. Moreover, clinical trials of our tool may provide further insights into its therapeutic potential in the treatment of osteosarcoma.

## Data Availability

All data and materials used in this study are available upon request.

## Abbreviations

CKM: Creatine kinase muscle
TERT: Telomerase reverse transcriptase
CRISPR–dCas9: Clustered regularly interspaced short palindromic repeats–deactivated Cas9
sgRNA: Single guide RNA
MDM2: Mouse double minute 2 homolog
